# Interactions between neural representations of the social and spatial environment

**DOI:** 10.1098/rstb.2022.0522

**Published:** 2024-09-04

**Authors:** James C. Thompson, Carolyn Parkinson

**Affiliations:** ^1^ Department of Psychology, and Center for Adaptive Systems of Brain-Body Interactions, George Mason University, MS3F5 4400 University Drive, Fairfax, VA 22030, USA; ^2^ Department of Psychology, University of California, Los Angeles, Los Angeles, CA, USA

**Keywords:** spatial navigation, social neuroscience, social networks, cognitive map, cognitive graph

## Abstract

Even in our highly interconnected modern world, geographic factors play an important role in human social connections. Similarly, social relationships influence how and where we travel, and how we think about our spatial world. Here, we review the growing body of neuroscience research that is revealing multiple interactions between social and spatial processes in both humans and non-human animals. We review research on the cognitive and neural representation of spatial and social information, and highlight recent findings suggesting that underlying mechanisms might be common to both. We discuss how spatial factors can influence social behaviour, and how social concepts modify representations of space. In so doing, this review elucidates not only how neural representations of social and spatial information interact but also similarities in how the brain represents and operates on analogous information about its social and spatial surroundings.

This article is part of the theme issue ‘The spatial–social interface: a theoretical and empirical integration’.

## 1. Introduction

When describing social information, people often use spatial language—describing others as ‘close friends’ and ‘distant strangers’, for example. Accordingly, it has been prominently argued that the pervasive use of spatial metaphors to describe social relationships (and other abstract information) is not merely an example of using metaphors for linguistic flourish, but rather, a reflection of how the mind represents such information [1]. Of course, people use many types of metaphors when speaking and writing, and simply using words from one domain of knowledge (e.g. space) to describe another (e.g. social relationships) does not necessarily imply deep similarities in processing or that conceptual mappings between domains are engaged when people think about such concepts in everyday life [2]. That said, at least some of the conceptual mappings between spatial and social information that pervade everyday language use can emerge in the absence of corresponding linguistic mappings (e.g. they have been documented in chimpanzees [3]). This is consistent with the possibility that such conceptual mappings between spatial and social information are not unique to humans and might reflect similarities in mental processing that inform linguistic mappings.

More generally, social and spatial phenomena are inherently interconnected. Geographic factors shape who we encounter, observe and befriend, as well as how we influence one another. Social factors shape how we perceive and explore the physical space around ourselves. Do these similarities run deeper than linguistic metaphors? And how exactly do spatial and social phenomena, as well as their underlying neural substrates, shape and constrain one another?

A growing body of research emphasizes commonalities and interactions between social and spatial phenomena for both humans and non-human animals. For example, the recently proposed Social–Spatial Interface framework [4] highlights that social and spatial behaviours share many concepts and terminology and that social and spatial phenotypes and environments interact with one another. Here, we examine a growing body of literature that highlights interrelations between social and spatial phenomena and their neural bases. We first discuss the neural basis of spatial cognition and introduce the topic of cognitive maps (§2). We then examine parallels in how the brain represents and reasons about social and spatial information and discuss the possibility that some types of social knowledge are encoded in cognitive maps and/or graphs akin to those used to represent space (§§3 and 4). Finally, we discuss how geographic factors impact social phenomena and vice versa (§§5 and 6).

## 2. Encoding spatial information and the cognitive map

A considerable amount of research into the neural basis of spatial navigation has focused on the role of the hippocampus. In primates, including humans, the hippocampus is located in the medial temporal lobe, and in rodents it is located between the neocortex and the thalamus [5]. Potential homologues to the mammalian hippocampus have been described in lizards [6], turtles [7], crocodiles [8], pigeons [9] and finches [10]. Evidence pointing to a role of the hippocampus in navigation came from studies showing that lesions to this region in rats and monkeys produced deficits in maze learning and other spatial tasks [11,12]. A correlation between amnesia and geographical disorientation in humans with brain injury was described by Benton ([13]; see also [14]). Perhaps the most well-described patient to undergo medial temporal lobe resection, Patient H.M. showed profound spatial disorientation and reportedly would frequently get lost soon after leaving his home [15]. Mammals are not the only species to show navigational deficits following lesions to the hippocampus. For example, lesions to the hippocampus of homing pigeons impaired their ability to navigate to their home loft [9].

### (a) The discovery and characterization of hippocampal place cells

**Figure 1.**
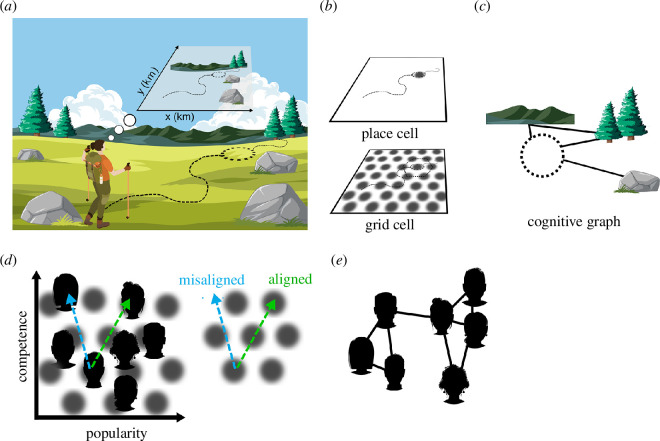
Cognitive maps and graphs for spatial and social information. (*a*) A cognitive map where any location is described by a set of two-dimensional Euclidean coordinates is thought to partially underlie mammalian spatial navigation. Here, a hiker’s cognitive map includes the path she travelled and various landmarks in metric coordinates. (*b*) Place cells represent specific spatial locations (place fields), whereas grid cells tile an environment using a hexagonal lattice. Both place and grid cells are thought to contribute to the cognitive map. (*c*) In cognitive graphs, only some locations (such as significant landmarks or places visited) are represented as nodes, and the paths between them are encoded as links (‘edges’). In this example, the hiker might connect the mountains, where she camped two nights ago, with the trees where she rested and ate lunch, and the location of her campsite last night. The rock is a less central landmark that she used to guide her path. (*d*) Some two-dimensional social attributes also appear to be represented in a cognitive map using a grid-like code. In the study by Park *et al*. [18], participants learned the attributes of competence and popularity of a group of individuals. fMRI data indicated that brain regions including the hippocampus and entorhinal cortex represented these individuals in a two-dimensional map. The researchers measured neural responses when participants considered how much a given individual would benefit from multiple potential collaborators based on their relative competence and popularity, which can be thought of as ‘thought trajectories’ in the two-dimensional cognitive map. Critically, trajectories aligned with the orientation of the grid pass through more grid cell firing fields (dashed green line) than trajectories that are misaligned with the grid (dashed blue line) and a macroscopic signature of this phenomenon is detectable with fMRI, providing a way to test if social knowledge is represented using a grid code. Park *et al*. [18] observed greater fMRI responses in the hippocampus, entorhinal cortex, IPC and MPFC when participants traversed ‘thought trajectories’ aligned with the two-dimensional grid (dashed green line) than when participants inferred trajectories between individuals not aligned with the two-dimensional grid (dashed blue line). (*e*) Some forms of social knowledge, including friendship networks, may be encoded using graph-like representations. IPC, inferior parietal cortex; MPFC, medial prefrontal cortex.

It was the discovery of hippocampal place cells, which selectively fire when an unrestrained rat is in a particular location of a testing platform ([16,17]), that cemented the role of the hippocampus in theories of spatial navigation. Cells recorded by O’Keefe *et al*. showed preferences for particular locations on the platform (named ‘place fields’), and when the rat was moved to a different location on the platform, a new set of cells began to fire. While the initial studies only recorded from a small number of neurons, subsequent research revealed that place cells were highly selective, and that each location visited by the rat was sparsely represented by the activity of only a few dozen cells [17]. It was suggested by O’Keefe [17] that from the activity of cells within the hippocampus, one could fairly easily decode where in the environment a rat was located. These place cells were argued by O’Keefe *et al*. to be the fundamental mechanism of a mammalian spatial navigation system (figure 1*a,b*).

Place cells are sensitive to large changes in the location of landmarks in the external environment, indicating a role of sensory inputs in forming place representations [16,19]. When moved to a new environment, individual place cells remap and represent new locations. On the other hand, most recorded place cells maintain their selective firing even when the rat is moving in darkness [20]. This suggests that path integration (self-movement) and allocentric environmental cues combine to form place representations [21]. In trying to understand the sources to place cells, researchers studied the inputs into the hippocampus. In mammals, cortical inputs to the hippocampus come via the entorhinal cortex. Recordings from medial entorhinal cortex (MEC) cells while rats freely explored an experimental enclosure revealed neurons that showed location-specific firing [22]. However, unlike hippocampal place cells, these cells in MEC displayed firing to multiple locations (figure 1*b*). Using a larger (2.2 m^2^) enclosure, Hafting *et al*. [23] observed that the spatial fields of MEC cells were organized in a hexagonal grid pattern, leading to them being named grid cells.

### (b) Grid cells in medial entorhinal cortex: an overview

The location (or phase), spacing (or wavelength) and orientation of grid cell fields vary from cell to cell [23]. This permits an almost complete spatial representation of the enclosure across a population of cells in MEC. In contrast to place cells, the firing of grid cells in MEC maintains a hexagonal grid structure across different environments [24]. Grid cell firing is also maintained across changes in the speed and direction of a rat as it moves through an enclosure, while distinct cell populations within MEC represent speed and heading direction [25,26]. These results have been argued to reflect an objective, context-invariant representation of spatial location based on path integration inputs [27]. While the hexagonal pattern of grid cells is maintained across different environments, sensory cues can influence the location, spacing and orientation of the grids. In particular, the boundaries of an enclosure can distort grid patterns [28], especially if they introduce an asymmetry to the environment [29], potentially leading to difficulties in using grid cell outputs as a direction signal.

The hexagonal structure of grid cell fields can be generated by a number of different biological mechanisms. A basic model involving a competition between short-range excitation and long-range inhibition can produce a hexagonal grid-like pattern like those observed in MEC grid cells [30]. Such hexagonal patterns have the advantage of being self-organized (i.e. they do not need an external control to form), are typically robust to external influences, and are an efficient way to represent two-dimensional data [31]. Continuous attractor models [32,33] and adaptation models [34] are both physiologically plausible models of MEC grid cell formation. It seems likely that grid cells provide path integration input to place cells, and there is growing evidence of projections from hippocampal place cells to MEC playing a role in grid representations [35]. In the simplest case, linear summation of multiple grid cell responses with similar location/phase, but different scales and orientations, would produce an activation peak at a single location consistent with a place cell response. However, experimental and anatomical data are inconsistent with a simple linear summation of MEC inputs into hippocampus place cells [36]. The flexibility and efficiency of hexagonal grid representations to encode spatial information, combined with tractable and biologically plausible models, make cells and/or populations of cells with these properties attractive as a possible general mechanism for the coding of low-dimensional information.

### (c) Delineating the neuronal basis of spatial navigation across mammalian species

Grid cells have been recorded in the entorhinal cortex of species other than rats, including mice [37], bats [38,39], non-human primates [40] and humans [41–43]. However, the spatial navigation system extends well beyond the hippocampus and entorhinal cortex. Within the parietal cortex, medial regions including retrosplenial cortex play an important role in encoding path integration and location in egocentric coordinates [44,45]. In the ventral temporal cortex, the parahippocampal region encodes scenes and the local arrangement of objects within those scenes [46]. In humans and other primates, multiple prefrontal regions, including the ventromedial and orbitofrontal cortex, dorsal anterior cingulate cortex and dorsolateral prefrontal cortex, encode navigational cues [41,47–49]. Grid cell responses have not been recorded outside the entorhinal cortex in rodents, although in humans, grid-like responses have been recorded in ventromedial prefrontal cortex [41] and anterior cingulate cortex [42]. Place cells have been harder to identify in human and non-human primates, perhaps owing to the difficulties in recording hippocampal neurons while participants move in an environment.

### (d) Constructing cognitive maps with grid and place cells

Together with place cells, grid cells have been proposed to provide a foundation for spatial navigation. Grid cells are argued to provide the navigating animal a metric of the distance to or from a reference location [27], while place cells provide details of location in world-centred coordinates [15]. Beyond the spatial domain, however, place cells and grid cells have been argued to provide the neural basis of a more general-purpose cognitive map [15,50–52]. There is evidence that behaviourally relevant dimensions of non-spatial tasks are coded in the hippocampus and MEC in a manner similar to the coding of space in these regions [53]. Constantinescu *et al*. [51] found that participants represented continuous, two-dimensional, non-spatial (conceptual) information in a cognitive map with a hexagonal grid-like code in entorhinal cortex and ventromedial prefrontal cortex.

### (e) Challenges for cognitive maps and alternative representational formats

While the classically described place and grid cells appear to provide a useful metric of space for navigation, and perhaps even the basis for more general cognitive maps, findings from naturalistic environments highlight a number of difficulties with this proposal. Place and grid cells were discovered using environments that were small, two-dimensional and spatially uniform, and the regularity of the hexagonal arrangement of firing fields in part reflects this uniformity. Many mammalian species, including rats in whom grid and place cells have been studied most intensively, navigate large-scale, non-uniform spatial environments, including open countryside, wooded areas and irregularly organized burrows. Complex and higher dimensional environments present a significant challenge to the creation of a grid-like arrangement of neuronal firing [36,39,54]. Even in spatially uniform environments, grid cells distort around locations of high motivational significance, such as the location of rewards or shelter [55,56]. Similarly, place cells over-represent the location of rewards [57].

One recent proposal is that rather than using a global Euclidean representation of space, we code space using topological or graph-like representations [36,52,58–60]. Unlike in cognitive maps, where each point in space has a corresponding set of coordinates, in cognitive graphs, only some locations, such as significant landmarks, are represented as nodes, and paths between them are encoded as links or ‘edges’ (figure 1*c*). Topological representations preserve the arrangement of elements within an environment, without requiring all details of the precise metric distances between elements. Hybrid topological-metric representations can even use a global topological representation to organize and represent local, metric maps that maintain precise spatial details [39,61]. Representations that preserve topology, rather than precise metric distance, might be better suited for the representation of non-uniform spatial data as well as non-spatial information, such as social information. As we discuss in the following section, there is growing evidence that we encode some aspects of social information within metric, grid-like cognitive maps. However, as is the case with spatial information, there are instances in which map-based representations are less suitable for encoding social information, and graph-based representations are more appropriate. We argue for the parallel use of maps and graphs in the representation of both spatial and social information, often engaging the same brain regions, consistent with the interconnected nature of these two systems.

## 3. Encoding social information: map- and graph-like representations in social cognition

A growing body of evidence has established correspondences between how people think about social and spatial information, as well as underlying neural mechanisms. More specifically, the hippocampal formation (i.e. the hippocampus and surrounding cortex) is most often implicated in the generation and use of world-centred (i.e. allocentric) cognitive representations, whereas the parietal cortex is most frequently implicated in encoding self-centred (i.e. egocentric) schemas that represent relational knowledge, including relational social knowledge [62], as described in more detail below. The use of brain structures and computational mechanisms with long-established roles in spatial cognition to process social information may reflect a more general extension of such substrates to process conceptual knowledge (including non-social conceptual knowledge). However, given the scope of this article, here we focus primarily on social information processing and its relationship to spatial cognition.

### (a) Egocentric representations of social knowledge

Much of everyday social cognition is inherently self-centred (i.e. egocentric). For example, one might need to retrieve knowledge about the closeness of one’s relationship with a particular person to inform decisions about how much to trust that person or to modulate the many aspects of cognition, affect and behaviour that are shaped by interpersonal closeness (e.g. attention, vicarious reward [63,64]). Such self-centred conceptual knowledge representations have been suggested to operate analogously to self-centred representations of physical space (i.e. representations of where and how far away things are relative to one’s body) and are consistently associated with brain regions within the parietal cortex [62]. For example, studies using fMRI have found that the inferior parietal cortex (IPC) encodes both proximity to oneself in physical space and social familiarity with other people [65]. This brain region appears to encode distance from oneself in space and social ties using a common coding scheme, such that neural response patterns signalling that someone is close to oneself in social ties or that an object is physically nearby are relatively similar, and neural response patterns signalling larger distances from oneself in either space or social ties are relatively similar [66].

That said, it is possible that the apparent common neural encoding of physical and social ‘distance’ from oneself reflects the encoding of a shared psychological meaning across domains (e.g. self-relevance or ‘psychological distance’) characterized by shared downstream consequences for cognition and behaviour (e.g. for construal level and attentional allocation [67]); and/or learned associations between social and physical closeness. Interestingly, other forms of spatial and social knowledge appear to be represented by distinct neural codes, even when they are often described using shared linguistic labels and are processed in overlapping areas of the parietal cortex (e.g. relative vertical positions in space and relative social status [68]). This is consistent with the notion that egocentric distance from oneself is a special case where social and spatial proximity have at least some shared meaning, and thus may be encoded in partially shared neural response patterns. In other cases, social and spatial information may be represented distinctly from one another, but still use shared brain regions and computational mechanisms. Such cases are likely much more common, and would include the representation of ‘cognitive maps’ of social knowledge.

In perhaps the first demonstration that the human brain encodes one’s social standing relative to others in a cognitive map of ‘social space’, Tavares *et al*. [69] had participants engage in a role-playing game during an fMRI study. This game involved virtual interactions with a set of characters who ‘moved’ in a social space defined by power and affiliation relative to the participant as a result of the participant’s decisions in the game. At any point in the game, each character’s position in this abstract social space could be described by a vector with a characteristic angle and length. Vector length (which captures the distance between a character and the participant in this abstract social ‘space’ defined by power and affiliation) was encoded in the precuneus and posterior cingulate cortex. The angle between the vectors describing the participant’s and character’s positions was encoded in a set of regions that included the IPC and hippocampus. These results are consistent with the possibility that people represent social knowledge using cognitive maps stored in brain regions that also map physical space, and that these maps facilitate social decision-making in an analogous way to how cognitive maps facilitate spatial navigation.

### (b) Allocentric representations of social knowledge

In everyday life, people often have to reason about abstract knowledge about other people. For example, one might need to determine who would be most qualified to provide advice about a particular topic or who would be the most trustworthy person to confide in regarding a delicate issue. Recent findings shed light on the neural mechanisms that support our ability to do this. Specifically, to represent and reason about other people’s traits [18], as well as other forms of abstract conceptual knowledge [51], the human brain appears to use low-dimensional cognitive maps analogous to world-centred representations of one’s spatial environment. Like world-centred representations of space, these abstract cognitive maps seem to involve the hippocampal formation [70], although similar representations have been observed in other brain areas, such as the posterior cingulate cortex, the IPC and the medial prefrontal cortex [18].

For example, Park *et al*. [18] had participants learn about two attributes (competence, popularity) characterizing a set of people who were ostensibly entrepreneurs. These entrepreneurs thus can be thought of as spanning a two-dimensional conceptual space where their positions are defined by their levels of competence and popularity. In a subsequent fMRI study, participants completed a task requiring them to consider the entrepreneurs’ competence and popularity to select ideal social partners. Entrepreneurs who were closer together in the two-dimensional conceptual space evoked more similar response patterns in several brain regions, including the hippocampus, entorhinal cortex, IPC and MPFC, consistent with the notion that knowledge about other people is represented as a cognitive map, analogous to cognitive maps of space (figure 1*d*).

Interestingly, this study also revealed deeper similarities between social and spatial processing. Specifically, Park *et al*. [18] found evidence for a grid-like encoding of social knowledge. To demonstrate this, they had participants perform mental operations analogous to an aspect of cognition that grid cells support: composing new routes and shortcuts [71]. Here, this involved generating new ‘thought trajectories’ in the conceptual space by considering how much a particular entrepreneur stood to benefit from one of two possible collaborators based on those collaborators’ levels of competence and popularity. In brain regions that encode such information in a grid-like code, ‘thought trajectories’ aligned with the grid would pass through more grid cell firing fields, and thus should evoke greater activity (figure 1*d*). The relatively coarse spatial resolution of fMRI precludes the ability to measure grid cell firing fields directly. However, the mean activity of a population of grid cells is higher for trajectories that are aligned with the main grid axes, which provides an indirect, macroscopic signature of grid-like coding that is detectable with fMRI. Specifically, regions containing grid cells should show a response profile characterized by sixfold rotational symmetry in the representational space, such that there should be six directions of ‘thought trajectories’ (each separated by 60 degrees in the representational space) that elicit equivalently strong responses. Park *et al*. [18] found evidence for grid-like coding of social knowledge in the entorhinal cortex, as well as in other regions, such as the temporoparietal junction, anterior temporal lobe, MPFC and superior temporal sulcus.

The encoding of two-dimensional social attributes seems well suited for a mechanism that uses a metric, grid-based cognitive map, engaging the same brain regions and—on the surface at least—similar coding mechanisms to the representation of spatial information. Compelling evidence suggests, however, that representations of other people’s mental states and actions are organized by more than two dimensions [72,73]. Whereas hexagonal grids are well suited to constructing precise two-dimensional maps, different geometric structures are ideally suited for representing higher-dimensional spaces (e.g. cubic lattices for three-dimensional spaces [74]). That said, much remains to be learned regarding how the brain actually encodes three-dimensional spaces, particularly given that grid cell firing appears to be relatively disordered and irregular when animals explore three-dimensional spaces, unlike the highly regular patterns observed when animals explore two-dimensional spaces [39,75]. In some cases, other non-map-based representational formats may be more appropriate. Relational social knowledge is also often multi-dimensional, dynamic and not metric, and might be better suited to graph-like coding mechanisms. Notably, the representational format that is evoked in a particular context may depend not only on the nature of the information at hand but also on characteristics of the paradigms used to assess those representations [60]. For example, when participants need to organize knowledge along two continuous dimensions, as in the Tavares *et al*. [69] and Park *et al*. [18] studies described earlier in this section, map-like representations are likely to be favoured [60]. On the other hand, when a task requires participants to reason about transitions between states/nodes, such as when reasoning about who is connected to whom in a social network, they may be more likely to use graph-like representations (figure 1*e*). In the following section, we describe the encoding of relational social knowledge, and highlight how the neural mechanisms that underlie this knowledge might be shared with those that represent aspects of spatial knowledge.

## 4. Encoding social networks

As described in the preceding section, mounting evidence suggests that shared neurocognitive mechanisms support representing and operating on social and spatial information. Such parallels with spatial cognition have been suggested to characterize how people think about any kind of relational social knowledge, and conceptual knowledge more generally [60,62]. Of course, relational social knowledge need not always pertain to social relationships *per se* (e.g. friendships). Any person knowledge (e.g. knowledge about others’ traits or preferences) becomes ‘relational’ when considering how different people compare to one another. Importantly, humans are keenly attuned to one particular kind of social knowledge that is inherently relational in nature: knowledge of the webs of social relationships that surround them.

### (a) The human social landscape: real-world social networks

Indeed, unlike many other social animals who interact with non-kin mainly in loose aggregations (e.g. swarms, herds), humans consistently form large, complexly bonded social groups composed of many long-term and intense bonds that often involve non-kin (e.g. friendships) [76]. Properties of individuals’ structural positions in such networks (e.g. the people to whom someone is and is not connected, how many connections someone has, whether or not someone ‘bridges’ between otherwise disconnected people or groups) have wide-ranging consequences for their own and others’ cognition and behaviour. For example, people who are closer together in a social network are more likely to cooperate with each other [77] and tend to be similar to one another in terms of diverse characteristics (e.g. age, gender, moral values [78,79]), to process the world similarly [80,81], and to evince similarities in brain structure and function [82,83]. Additionally, people who are particularly well-connected in their social networks tend to process the world in ways that are particularly reflective of norms in those networks [84], tend to be protected from negative gossip and maltreatment [85] and are able to spread information exceptionally effectively [86]. Such individuals also evince distinctive patterns of brain connectivity in neural systems that support social and affective processing [87] suggesting that much like trait-level differences in capacities relevant to navigating and managing physical space can shape and be shaped by one’s physical habitat, trait-level differences in the brain networks that support navigating one’s social environment (e.g. affective processing, social perception, understanding others’ actions) may shape the ‘positions’ that individuals end up occupying in their social networks.

### (b) People acquire, integrate and use nuanced knowledge of their social networks

Achieving success, maintaining harmony and avoiding conflict require that people not only track and manage their own relationships with others; they also must monitor and remember relationships among others (e.g. who is friends with whom) and patterns thereof (e.g. who is well-connected, the existence of cliques). Learning the structure of these networks, as well as non-social networks, is supported in part by the hippocampus [88]. Just as people navigate space using somewhat accurate, but somewhat distorted and schematic, spatial representations [89,90], people maintain somewhat accurate, although imperfect, mental representations of their social networks [91]. From early on in childhood, humans pay attention to interactions and apparent relationships between third parties, which can help to construct such social cognitive ‘maps’, and use such knowledge to shape their social inferences and behaviour (e.g. to determine who knows what [92]; to inform interpretations of gossip [93]). Information about patterns of social relationships between others appears to be so important to humans that when people think about or encounter others, or even merely view familiar others’ faces, our brains spontaneously encode a rich set of information about those individuals, including aspects of where those individuals ‘sit’ in social networks [94–100].

After retrieving knowledge of others’ positions in their social networks, people appear to use this information to shape many different aspects of their cognition and behaviour. For instance, when one only has incomplete or ambiguous information about another person, as is often the case in everyday life (e.g. with new acquaintances), knowledge about that person’s social ties can inform predictions about that individual. For example, people tend to assume that others will share traits and preferences with their friends [101,102]. Such assumptions shape subsequent behaviour—e.g. people are less likely to trust those who are known to have untrustworthy friends, even when they consistently show themselves to be trustworthy [101]. Similarly, people appear to be aware of the fact that those who occupy central positions in their social networks are particularly likely to behave in ways that are reflective of community norms, as they preferentially look to such individuals to efficiently ascertain social norms [103]. In addition to using social network knowledge to guide inferences about others, people can also apply such knowledge to guide effective information-sharing [86], manage their own reputations (e.g. showing more positive attributes when being observed by central others [104]), and manipulate their social environment, such as by participating in defensive behaviours aimed at protecting one’s own friendships, avoiding replacement by others, and maintaining one’s social standing (e.g. [105,106]).

### (c) How do people build mental representations of their social networks?

Clearly, people are very attuned to information about others’ locations in their social networks and use such knowledge in many aspects of everyday life. How do people acquire such knowledge in the first place? Tracking relationships between others in a large social group is a demanding endeavour. Even setting aside more complex aspects of social network knowledge, simply tracking the dyadic relationships in a social group (e.g. who is friends with whom) can be quite computationally demanding, as the number of possible relationships increases exponentially with group size. Thus, people rely not only on their own direct observations but also on others’ observations, which are often conveyed via gossip, to learn about relationships and interactions between third parties [107]. However, even with the aid of others’ observations, it is likely typically infeasible to monitor all possible relationships between others in one’s network. Thus, people incorporate an arsenal of schemas and heuristics to ‘fill in the gaps’ in their knowledge of social relationships.

Some such heuristics are relatively simple. For example, just as expectations of homophily (i.e. the tendency for similar others to affiliate with each other) serve as a ‘social prior’ that people apply when using friendship knowledge to ‘fill in the gaps’ of their knowledge about what other people are like, people also use knowledge of similarities in others’ traits and preferences to predict who is friends with whom [101,102,108]. Relatively simple heuristics can also inform inferences about others’ centralities in social networks. For instance, people use others’ apparent personality traits (e.g. agreeableness, extraversion) and other appearance information (e.g. apparent gender, attractiveness) to predict the likely social network centralities of unfamiliar others [109,110]. People could also apply other simple shortcuts, such as assuming that people whom they hear about more often are better-connected, to ascertain others’ likely centralities in their networks [86]. Such simple heuristics can substantially reduce the information and effort required to infer information about others’ positions in social networks.

Other strategies that people use to ‘fill in the gaps’ in their social network knowledge are somewhat more nuanced and sometimes mirror how people think about non-social (e.g. spatial) information. For example, recent evidence suggests that when attempting to recall the structure of social networks, people tend to focus on a small number of well-connected (i.e. high-centrality) individuals and connections involving those individuals [111]. This in some ways resembles landmark-based wayfinding, a strategy that humans and other animals use to navigate space [112] and is also analogous to proposals that route-based spatial navigation is facilitated by cognitive graphs in which only a small number of significant locations are represented as nodes [113]. Interestingly, the retrosplenial complex, a brain region that has long been implicated in spatial processing, and in particular, in representing physical locations in space [112], encodes information about others’ positions relative to each other in a perceiver’s real-world social network, further suggesting the possibility that social and spatial relations are processed similarly [98].

Other recent work has extended perspectives from the literature on cognitive graphs to explain how people make principled inferences about unobserved social ties among others [114]. More specifically, people may use a multi-step relational abstraction mechanism that involves combining knowledge of direct (e.g. friends) and indirect (e.g. friends-of-friends) relations to generate and update graph-like mental representations that encode the likelihood that each pair of individuals in a network will be observed together. Interestingly, many systematic biases in people’s mental representations of social networks could be driven either by a collection of learned schemas and heuristics (e.g. friends are likely to be similar; people are likely to be friends with their friends’ friends) or could naturally emerge from probabilistic inferences based on such cognitive graphs [114].

While recent work has made great progress in advancing understanding of how people acquire and use knowledge of the structure of their social networks, much remains to be learned. Several studies highlight parallels in how people learn, represent and reason about social and spatial knowledge, as well as the neural basis of these processes. Given that our understanding of how the brain represents and navigates space is comparatively more advanced than our understanding of analogous social phenomena, future work could benefit from applying perspectives and methods that have been used to study spatial cognition to reveal further insights into how people learn about, encode and use knowledge of the structure of their social networks.

## 5. Spatial constraints on social networks

In addition to the parallels between the mental processing of social and spatial information highlighted in the preceding sections, social phenomena are also fundamentally related to, and often constrained by, spatial factors. Indeed, geography is a powerful determinant of our social relationships, shaping everything from the people we meet, to the extent to which homophily and social influence processes can unfold and shape our social networks, to the relationships that we can feasibly maintain.

### (a) Geographic distance shapes the relationship between neural similarity and social distance

Perhaps the simplest way that spatial factors impact social networks is by determining who we meet. Geographic proximity shapes who we encounter and befriend in our day-to-day lives [115,116]. As a result, geographic distance moderates the relationship between interpersonal similarity and social distance (i.e. how many ‘degrees apart’ two people are) in a social network. For example, in one study that examined interpersonal similarity in patterns of brain connectivity at rest (i.e. ‘functional connectomes’), the geographic distance between people’s homes and the distance between them in their social network, the relationship between neural similarity and social distance was strongest among people who lived closest to one another, and the strength of this relationship decreased with greater geographic distance between people’s homes [82].

This phenomenon could be driven by the constraints that geographic distance exerts on both homophily and social influence processes. With respect to homophily—i.e. the tendency of already-similar people to associate with one another—people who live close to each other are likely to have more chances to encounter each other, and thus befriend each other if they determine that they are compatible. On the other hand, we are less likely to encounter people who live far from us, and thus, such people are likely to be a mix of individuals with whom we would and would not be compatible as friends. With respect to social influence, geographic distance similarly constrains processes that could otherwise engender links between social network proximity and interpersonal similarity. More specifically, geographic distance between people limits the opportunities that those individuals have to observe one another’s behaviour and to interact with one another, and thus, to influence one another. Thus, the strength of the relationship between interpersonal similarity and social distance decreases with geographic distance, likely because homophily and social influence processes play out less readily among people who are significantly spatially removed from one another.

### (b) Neurocognitive limitations on technology-mediated expansion of social networks

It is possible that the constraints that geographic distance places on human social networks will be dampened as online social networks and technology-mediated social interactions (e.g. mobile phone conversations, video chatting) become more prevalent. Yet, in contrast to many people’s intuitions, use of online social media does not appear to have much impact on the size and range of human social networks [117,118]. This may be owing to several factors.

First, large-scale studies of technology-mediated social behaviours suggest that people most frequently use technological tools (e.g. mobile phones) to connect with those in close geographic proximity to themselves, using their devices primarily to supplement interactions with those who they already regularly see face-to-face, rather than to connect with people who live far away [119]. Second, limits on available time constrain the number of relationships that people can form and maintain, particularly for close relationships, which require significant investments of time on the part of those involved [120–123]. Third, and of particular relevance to the current article, there are also significant neurocognitive constraints on the number of social relationships that people can maintain, owing in part to humans’ limited memory capacity [124]. Similarly, several studies have demonstrated links between the size and structure of key brain regions involved in social cognition and number of social contacts [125–128], and at least in some cases, this relationship appears to be driven by social cognitive competencies such as mentalizing [126].

Thus, the same neural and cognitive capacities that allow humans to form social networks also constrain the size of those networks. These neurocognitive constraints, along with other limiting factors (e.g. constraints on available time) and tendencies (e.g. to use technology to reinforce relationships with physically proximal others) likely contribute to social networks remaining about the same size with and without the use of technology that can facilitate connection between people in remote physical locations.

## 6. Social influences on spatial processing

As highlighted in the previous section, social relationships are heavily influenced by spatial factors such as geography. The interactions between social and spatial processing can flow in the opposite direction as well, as social concepts can modify how individuals mentally represent and interact with the physical space around themselves.

### (a) Conceptual knowledge distorts mental representations of space

Conceptual representations can influence metric representation of space in a number of ways. Encoding irregular spatial environments can be particularly challenging, and we appear to use heuristics to facilitate spatial encoding and inference [129]. When there is no strong objective frame of reference, individuals tend to adopt stereotypical shapes as reference frames, and rotate their representation of the configuration of elements within the reference frame accordingly. Similarly, we tend to expect perceptual figures that are close together to align with each other. As a result, individuals can make systematic errors in the spatial location of geographical features [129]. For example, individuals in the USA often mistakenly judge the city of Detroit to be west of Atlanta, as the former is usually considered part of the midwest and most of Michigan is west or Georgia, while northern Europeans mistakenly report that Gothenberg is east of Copenhagen, as most of Sweden is east of Denmark [130].

### (b) Social concepts distort mental representations of space

Social concepts can also influence spatial judgments and introduce systematic distortions of geographical features. In one study, Burris and Branscombe [131] showed that participants from the US systematically overestimated the distance between two cities that lie on different sides of the US/Canadian border, relative to two cities within the US. A similar effect of the US/Canadian border was observed in Canadian participants, who judged an American and Canadian city as further apart than two Canadian cities separated by an equivalent distance. These effects did not appear to be just the result of a contiguity bias, as US participants judged a city in Alaska to be closer than non-US cities, even when the physical distance from the reference was similar. Participants did not show these same distortions of distance when judging distances between two different foreign locations [131]. These findings suggest that self-relevance is important to how social concepts influence spatial judgments. Consistent with this suggestion, Maddox *et al*. [132] demonstrated how the socially relevant concept of race can influence spatial inferences. Participants learned the location of a number of features in a fictional city. Many of these locations were associated with different racial groups. For example, some businesses were described as primarily serving Black or White customers and a dry cleaning business was described as having an owner with an Asian-American name. Features were clustered into neighbourhoods that were largely segregated by race. After learning the locations of features and their neighbourhoods on a map, participants then had to make estimates of the distance between features. Participants systematically overestimated the distance between features that were located in different racially defined neighbourhoods. A follow-up experiment found that such effects were not present when a less-salient social concept, political affiliation, was used to define neighbourhoods. The neural mechanisms that underlie the influence of social information on spatial representations have not been studied extensively. This is an area that we hope future research might examine to provide new insight into the interactions between social and spatial cognition.

### (c) Social factors shape interactions with the spatial environment

The presence of others and the membership of a social group can influence spatial behaviour beyond estimates of location or distance. The influence of group membership on spatial behaviour, including foraging for food, in non-human animals has been well described [133]. In particular, being part of a group can help protect against predators and other threats. In humans, the presence of other people can reduce the perceived threat of a foe [134], and participants expecting an aversive outcome, such as a painful shock, will choose to be in a group versus being alone [135]. Tedeschi and colleagues [136] found that membership of a group decreased fear from threatening stimuli, with an increasing effect as a function of group size. Consistent with behaviour observed in non-human species, humans will trade-off predation risk and reduced reward availability owing to the presence of others when engaged in foraging behaviour. Silston *et al*. [137] found that when performing a computerized patch foraging task with patches that varied according to the threat of capture by a ‘predator’ and the presence of others in the patch, participants weighed both these variables to determine perceived patch value. In the absence of a threat, participants preferred the patches that had fewer competitors for resources. However, when the threat was present participants were more likely to choose the highly inhabited patches. Using fMRI, they found that perceived patch value (including the combination of threat and number of competitors) was neurally represented in MPFC and the hippocampus, suggesting these regions incorporate social and threat information when representing the reward value of a spatial location.

## 7. Conclusion: interactions at the neural social–spatial interface

We have argued that links between spatial and social phenomena go beyond linguistic metaphors, and reflect deeper interconnection between the representation of these categories of information. There is growing evidence that map-based representations are used to code two-dimensional spatial and social information, and there appears to be considerable overlap in the neural basis of these representations within the hippocampus, entorhinal cortex, retrosplenial cortex and ventromedial prefrontal cortex. Maps work well for low-dimensional data, but it is not clear if they are used to represent non-uniform, clumpy, high-dimensional data. Cognitive graphs might reflect a parallel mechanism that works alongside grid/map-based representations, depending on the environment and the goal of the task. The overlap in the neural basis of social and spatial representations, and evidence of both domains employing similar representational formats, might suggest the operation of a domain-general neural mechanism. In this review, we have chosen not to provide a single conclusive answer to the question of whether or not social and spatial knowledge are subserved by a common underlying neural mechanism. This is because the answer to this question, and the set of neural mechanisms that are brought to bear on any social or spatial task, depends critically on the precise nature of the social or spatial information in question, as well as the structure of the task at hand. Instead, we highlight multiple instances of striking commonalities in how the brain supports the encoding and navigation of social and spatial knowledge and the extensive ways in which the social and the spatial interact. Given the commonalities and interactions outlined in this review and the other articles in this theme issue, we hope that the reader will come away from this issue appreciating how closely linked these two domains are. It is also important to consider that in some cases, apparent overlap in neural and representational mechanisms for spatial and social knowledge might also at least partially reflect the difficulty of fully disentangling the social from the spatial (and vice versa), even if distinct mechanisms, at the level of neural processing, are responsible for each domain. Future research is needed to resolve these lingering questions, and we hope that this article, and the Social–Spatial Interface framework in general [4], might help inspire such research. There are many other promising directions for future research into the interconnections between spatial and social cognitive neural representations, including understanding in greater detail how geography constrains social representations, how technological advances influence both spatial and social behaviour, and how social concepts can modify our neural representations of space. In highlighting the connections between the two rich areas of research, we hope that findings from each area can help inform our understanding of, and develop new hypotheses about, the other.

## Data Availability

This article has no additional data.
